# NPLOC4 is a potential target and a poor prognostic signature in lung squamous cell carcinoma

**DOI:** 10.1038/s41598-023-47782-6

**Published:** 2023-11-22

**Authors:** Naixue Wang, Dantong Zhu, Yao Liu, Jingran Wu, Meiling Wang, Shanxiu Jin, Fangwei Fu, Baolei Li, Hongjuan Ji, Cheng Du, Zhendong Zheng

**Affiliations:** 1 Department of Oncology, General Hospital of Northern Theater Command, Shenyang, 110011 China; 2 Jinzhou Medical University, Jinzhou, 121001 China; 3 Dalian Medical University, Dalian, 116085 China; 4 Shenyang Pharmaceutical University, Shenyang, 110016 China; 5 Guizhou Medical University, Guiyang, 550025 China

**Keywords:** Biochemistry, Cancer, Cell biology, Computational biology and bioinformatics, Immunology, Biomarkers, Oncology

## Abstract

Few prognostic biomarkers exist for lung squamous cell carcinoma (LUSC), which has a poor five-year survival rate. Using bioinformatics, this study evaluated NPLOC4 as a prognostic marker for patients with lung squamous cell carcinoma. Shorter survival periods and tumor growth were linked to high NPLOC4 expression.Disulfiram (DSF) combined with copper (Cu) targets NPLOC4 to achieve antitumor effects in lung squamous cell carcinoma. Thus, we investigated the effects of DSF with Cu in LUSC. Gene-set enrichment analysis identified ubiquitin-mediated proteolysis as the NPLOC4-associated mechanism influencing LUSC prognosis. In SK-MES-1 cell lines, DSF + Cu increased K48-linked ubiquitinated protein expression and apoptosis. This study identified NPLOC4 as a prognostic biomarker and a potential therapeutic target for LUSC.

## Introduction

Although lung squamous cell cancer (LUSC) accounts for only 15% of the lung cancer cases, more than 315,000 new cases are diagnosed each year^1^. LUSC has a poor prognosis^2^ with a five-year survival rate of less than 20% for patients with inoperable LUSC^3^ and limited treatment options. The nuclear protein localization protein 4 gene (NPLOC4), encodes NPLOC4, a protein that maintains nuclear membrane integrity and is involved in nuclear pore transport^4^. NPLOC4 is composed of an Ubx-like (UBXL) domain, zinc-finger domain, Mpr1/Pad1 N-terminal domain, C-terminal domain, and NZF domain^5^. Previous studies have clearly shown that NPLOC4 is involved in the ubiquitin–proteasome pathway^6–9^ and is essential for the ubiquitin-mediated degradation of a diverse group of ER proteins^5^.More importantly, Skrott et al.^10^ showed that NPLOC4 inhibition can lead to polyubiquitinated protein accumulation, which can induce cell death. Previous studies suggest that a combination of disulfiram (DSF), an anti-alcoholism agent^11^, and copper (Cu) could kill tumor cells by acting on NPLOC4^10,12–14^. Given its biological properties NPLOC4, it is a promising potential target and biomarker.

In this study, we analyzed differential transcriptional and proteomic gene expression, as well as potential biological interaction networks, to evaluate the prognostic value of NPLOC4 as an oncogenic gene in LUSC. We also investigated the cytotoxic effect of DSF combined with Cu on lung squamous carcinoma cells and its potential molecular mechanism. We aimed to identify a new prognostic biomarker and potential therapeutic targets for LUSC.

## Materials and methods

### Data source

Lung squamous carcinoma transcriptional and pro data were downloaded from The Cancer Genome Atlas (TCGA) database (www.portal.gdc.cancer.gov). Additional data were obtained from the LUSC tissue microarray (TMA, HLugS180Su01), purchased from Shanghai Outdo Biotech Company (Shanghai, China). These tissues were pathologically diagnosed as squamous lung cancer.

### Immunohistochemistry

NPLOC4 antibody (ab 224,435; Abcam) was used for immunohistochemical evaluation of NPLOC4. Immunohistochemistry kits were purchased from ZSGB-BIO (China, no: PV-91000). Tissue sections were dewaxed, antigen-repaired for 2 min 15 s at 120 °C, and blocked with peroxidase blockers. After overnight incubation with NPLOC4 antibody (1:45) at 4 °C, reaction enhancement solution was added to the sections. Secondary antibody was then added and incubated at room temperature. Finally, the tissue sections were reacted with 3,3‐diaminobenzidine and counterstained with hematoxylin. Immunohistochemical staining intensity was categorized as 0, negative; 1, weak; 2, moderate; 3, strong; or 4, stronger. The protein expression fraction was calculated as the ratio of the number of positive cells to the total number of cells, which was then multiplied by the staining intensity score to obtain a total score. For each sample, three random images with different perspectives were evaluated and the average score calculated.

### Differentially expressed gene analysis

We obtained UCSC Xena (https://xena.ucsc.edu/) NPLOC4 expression data from 33 tumor types, converted the data to standardized transcript-per-million format and used the "ggpubr" and "ggplot2" R packages to draw box plots of NPLOC4 expression in the 33 tumors. The NPLOC4 expression profile was extracted from transcriptomic data of the downloaded LUSC dataset, and NPLOC4 expression levels in cancer and paraneoplastic tissues were analyzed using the "Limma" package in R v3.6.3 using scatter plot difference analysis. NPLOC4 expression in cancer and para-cancer tissues of each patient was compared using paired difference analysis.

### Survival analysis

Survival analysis of patients with LUSC was performed using the Oncolnc online database (http://www.oncolnc.org/). Univariate and multivariate Cox regression analyses were used to assess the effects of clinical variables on patient outcomes. Receiver operating characteristic (ROC) curves were plotted using the “pROC” and “ggplot2” R packages. A time-dependent ROC curve was constructed using the “timeROC” package to evaluate predictive accuracy.

### Protein–protein interaction network construction

We constructed a network of proteins associated with NPLOC4 using the STRING database (https://cn.string-db.org/). Nodes with a confidence of interactive relationships larger than 0.4 were used for building the network. Visualization of protein networks using cytoscape software.

### Gene enrichment analysis

Based on the median NPLOC4 expression, tumor samples were divided into high-expression and low-expression groups. Whole gene expression data from two groups of patients were substituted into GSEA v4.1.0, and the number of iterations were set to 1000. A KEGG dataset was selected to explore the possible mechanism of the effect of NPLOC4 on pulmonary squamous carcinoma prognosis. Differentially enriched pathways were scored, with higher scores for significant enrichment in the higher-risk group, and lower scores for significant enrichment in the lower-risk group. Enriched pathways were filtered based on *P*-values (< 0.05) and FDR values (< 0.05).

### Correlation of NPLOC4 expression with immune cell infiltration and immune checkpoints

To analyze the relationship between tumor-infiltrating immune cells (TIC) and NPLOC4 expression, the distribution of TIC abundance in tumor samples was estimated using CIBERSORT to select tumor samples with *P* < 0.05. Correlation between NPLOC4 expression and immune checkpoints was evaluated using the TIMER database (https://cistrome.shinyapps.io/timer/).

### Cell lines and reagents

SK-MES-1 and BEAS-2B were purchased from Shanghai Collection Cell Bank of the Chinese Academy of Sciences. SK-MES-1 was cultured in MEM (Meilunbio, Dalian, China) supplemented with 10% fetal bovine serum (FBS; Biological Industries/Sartorius, Cromwell, CT, USA) and 1% penicillin/streptomycin (HyClone, Logan, Utah, USA) at 37 °C in a humidified atmosphere with 5% CO2. BEAS-2B was cultured in DMEM (Thermo Fisher Scientific, Waltham, MA, USA) supplemented with 10% fetal bovine serum at 37 °C in a humidified atmosphere with 5% CO2. Free DSF and Cu gluconate (Sigma-Aldrich, St. Louis, US) were dissolved in dimethyl sulfoxide (DMSO, Sigma-Aldrich, St. Louis, US) and stored at 100 mM at 4 °C.

### Cell viability assay

Cck8 kits were purchased from NCM Biotech (Soochow, China) to measure cell viability. SK-MES-1 cells were seeded onto 96-well plates (12,000/well) and, following cell adherence, treated with DSF, Cu, or a DSF + Cu combination. After 24 h, CCK-8 reagent was added to the wells and cell viability assessed according to the manufacturer’s instructions. Cell survival was 50% at 0.25 μM DSF and 0.2 μM Cu, and these concentrations were used for subsequent experiments.

### Western blot analysis

SK-MES-1 cells were treated with 0.25 μM DSF and/or 0.2 μM Cu for 24 h. The cells were then lysed in a mixture of RIPA buffer and PMSF (1000:1), followed by protein purification. Extracted proteins were quantified using a BCA protein assay kit (Bioss, Woburn, MA, USA). Target proteins were separated by electrophoresis on 10% sodium dodecyl sulfate–polyacrylamide gels (Bio-Rad, Hercules, CA, USA) using 15 μg protein sample per well. The separated proteins were transferred to nitrocellulose membranes (Bio-Rad, Hercules, CA, USA) and blocked for 10 min at room temperature using blocking solution (Genefist, Shanghai, China). The transfer membranes were subsequently incubated with the prepared primary antibodies (anti-NPLOC4,1:2500 Abcam, cat. no: ab224435; anti-K48-ubiquitin 1:10,000, Abcam, cat. No: ab140601; anti-β-actin1:5000, Abcam, cat. no: AF7018) overnight at 4 °C and then incubated with the secondary antibody (Proteintech, Chicago, USA) for 2 h at room temperature. Finally, ECL reagent (Bio-Rad, Hercules, CA, USA) was added to the transfer membrane, incubated, and images captured using a Versa Docimaging System.

### Apoptosis detection

An in situ cell death detection kit (Roche, Basel, Switzerland) was used to detect apoptosis. Cells were treated with 0.25 μM DSF or 0.2 μM Cu for 24 h and cell death evaluated according to the manufacturer’s instructions. Treated cells were photographed using a Nikon fluorescence microscope (Nikon, Washington, USA).

### Statistical analysis

Differential expression of NPLOC4 in cancerous and paraneoplastic tissues in the same sample was analyzed using the Wilcoxon nonparametric test and paired *t*-test. Survival analysis was performed using the Kaplan–Meier method with the log-rank test. *p* < 0.05 was considered significant. Univariate and multivariate Cox regression analyses were used to assess the effects of clinical variables on patient outcomes. All experimental data are presented as the mean ± standard deviation of at least three independent experiments. Statistical analyses were performed using R software v3.6.3 and GraphPad Prism 7.0.

### Ethics approval

The study was approved by the ethics committee of Shanghai Outdo Biotech Company (ID:YB M-05–02).

## Results

### Differential expression of NPLOC4 and association with clinicopathological variables

**Figure 1:**
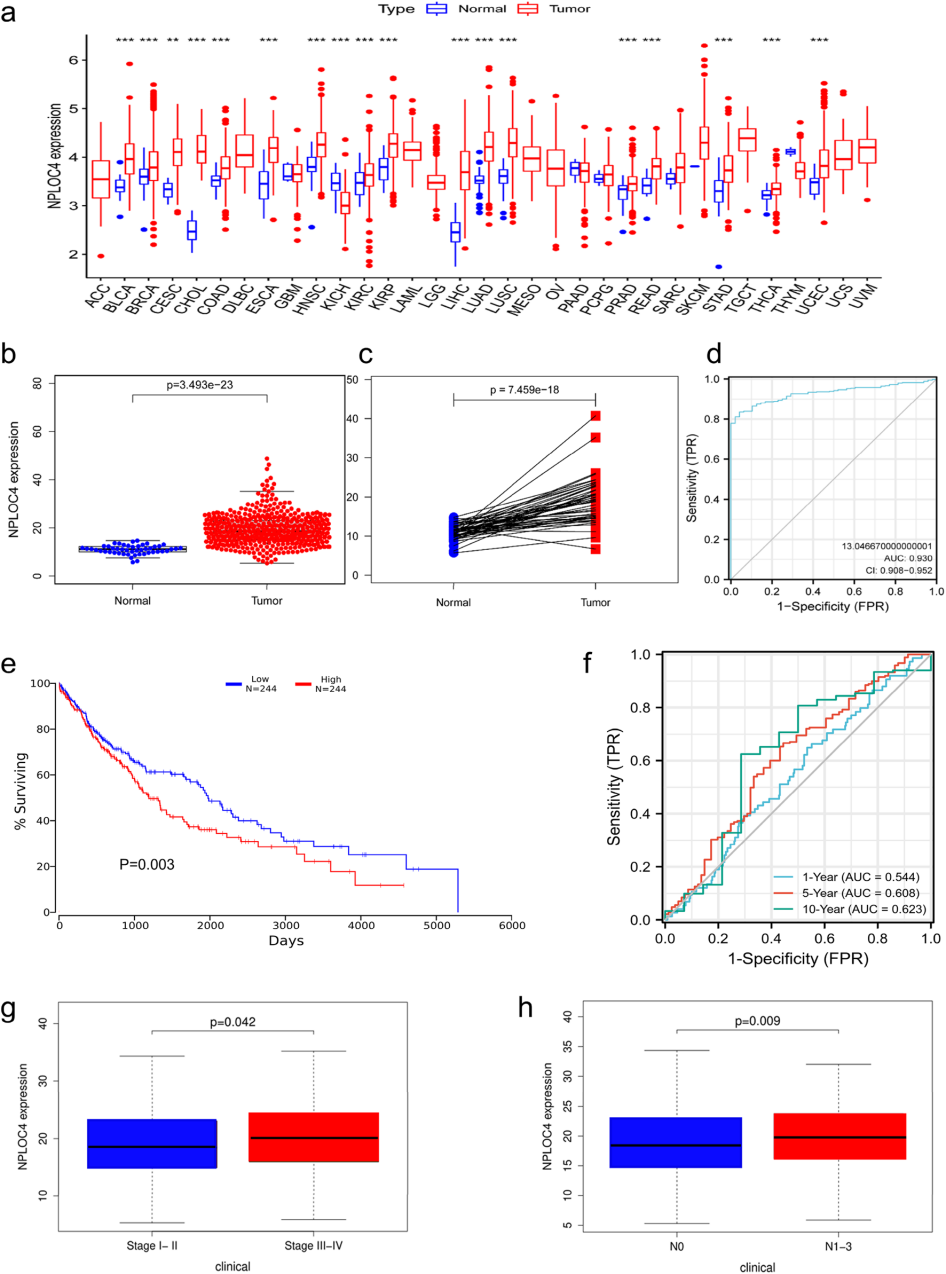
(**a**) NPLOC4 expression in different types of tumors compared with normal tissues in TCGA database. NPLOC4 expression in (**b**) LUSC tissues and normal tissues in TCGA database, and (**c**) matched normal tissues in TCGA database. (**d**) ROC curves for classifying breast cancer versus normal breast tissue in TCGA database. (**e**) Survival curves show that high NPLOC4 expression in patients with LUSC is strongly associated with shorter overall survival (OS). (**f**) In TCGA dataset,time-dependent ROC curves predict patient survival at 1, 3, and 5 years according to NPLOC4 expression. Associations between NPLOC4 expression and clinicopathological characteristics. Data are shown for (**g**) clinical stage, (**h**) N stage.

Pan-cancer analysis indicated that NPLOC4 was highly expressed in most types of cancer, including clear cell renal cell carcinoma, bladder urothelial carcinoma, and papillary renal cell carcinoma (Fig. 1a). NPLOC4 expression was significantly higher in LUSC samples than in normal lung tissues (*p* < 0.001) (Fig. 1b). NPLOC4 was highly expressed in 49 samples of lung squamous carcinoma tissue (*p* < 0.001) (Fig. 1c). The ROC curve showed that NPLOC4 expression could discriminate between LUSC tissues and normal lung tissues with an area under the curve (AUC) of 0.930 (95% confidence interval [CI] = 0.908–0.952) (Fig. 1d). Kaplan –Meier survival curves showed that patients with high NPLOC4 expression had shorter survival times, while those with low expression had longer survival times (Fig. 1e). Validation of the total survival model using ROC curves (Fig. 1f) showed AUCs of 0.544, 0.608, and 0.623 for 1, 5, and 10 years, respectively. To determine the relationship between NPLOC4 expression and clinicopathological characteristics (Fig. 1g,h and Supplementary Fig. 1), we analyzed the corresponding clinical information of LUSC cases from the TCGA database. We found that patients with TNM stages III–IV, as well as patients with lymph node metastasis, had higher NPLOC4 expression. The elevated NPLOC4 expression may be associated with LUSC migration and invasion.

### NPLOC4 expression in TMA and clinical sample validation using the TMA database

**Figure 2:**
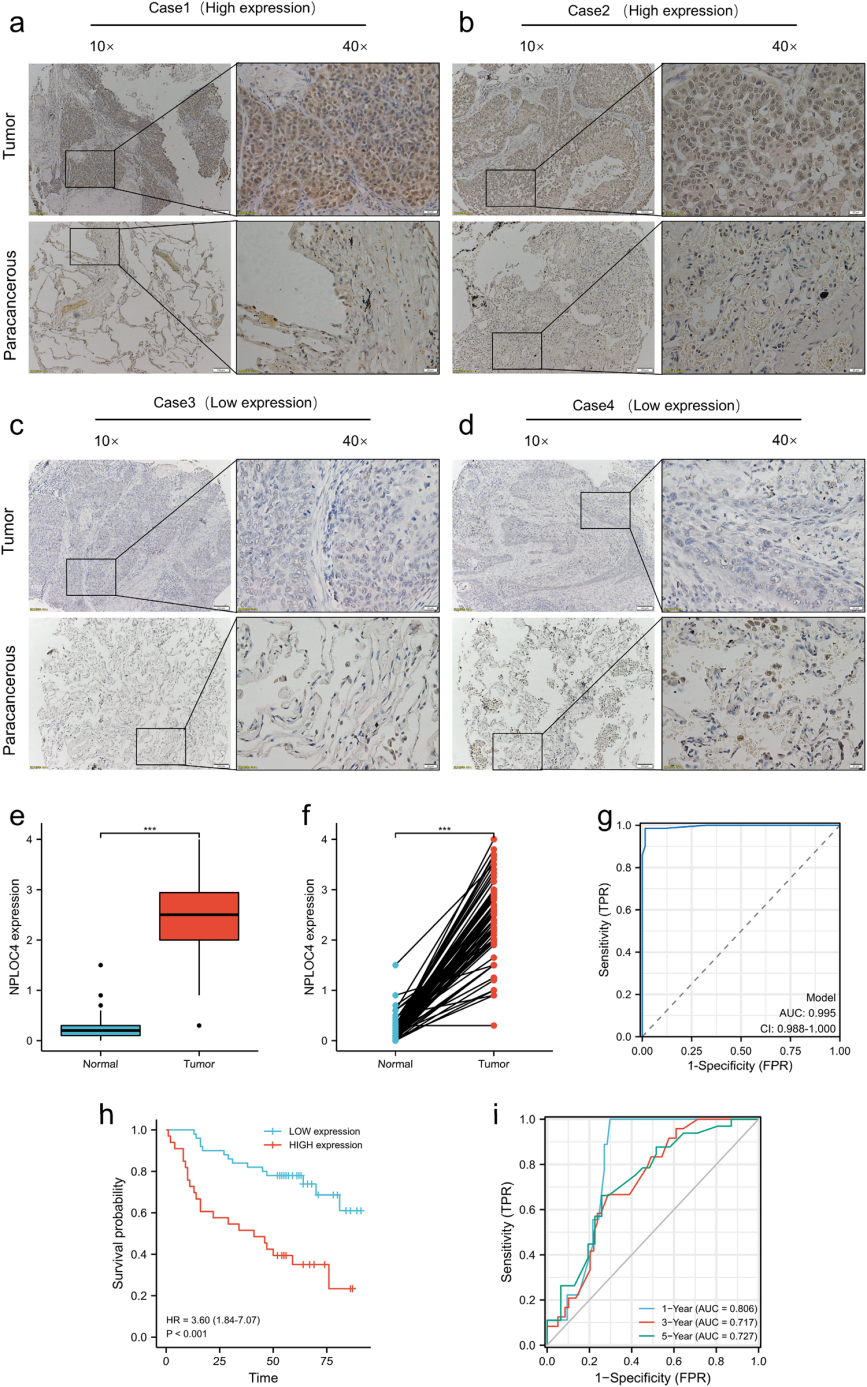
NPLOC4 expression in cancer and para-cancer tissues. Original magnifications 10 × (Scale bar: 100 µm.) and 40 × (Scale bar: 20 µm.) ,inset panels. (**a**) Case 1 and (**b**) Case 2 exhibit high NPLOC4 expression in cancer tissues. (**c**) Case 3 and (**d**) Case 4 exhibit low NPLOC4 expression in cancer tissues. In the TMA database, NPLOC4 expression in (**e**) cancer and normal tissues, and (**f**) matched normal tissues. (**g**) ROC curves for classifying breast cancer versus normal breast tissue in TMA database. (**h**) Survival curves of the high and low NPLOC4 expression groups. (**i**) Time-dependent ROC curves predict patient survival at 1, 3, and 5 years according to NPLOC4 expression.

To further validate NPLOC4 expression, immunohistochemical staining was performed on 90 cancer and para-cancer tissue samples. Data was obtained from 83 patients. Cases 1–4 are representative of NPLOC4 immunohistochemical staining. Cases 1 (Fig. 2a) and 2 (Fig. 2b) exhibited high NPLOC4 expression in cancer tissues. Cases 3 (Fig. 2c) and 4 (Fig. 2d) exhibited low NPLOC4 expression in cancer tissues. Evaluation of NPLOC4 expression in conjunction with clinicopathological data from 83 patients revealed higher NPLOC4 expression in tumor tissues, both in the analysis of cancerous and paraneoplastic tissues (Fig. 2e) and in the paired analysis of cancerous and paraneoplastic tissues (Fig. 2f). The ROC curve showed that NPLOC4 expression could discriminate between LUSC and normal lung tissues with an area under the curve (AUC) of 0.995 (95% confidence interval [CI] = 0.998–1) (Fig. 2g). Kaplan–Meier survival analysis revealed that patients with high NPLOC4 expression had shorter survival times than those with low expression (Fig. 2h). Validation of the total survival model using ROC curves (Fig. 2i) showed AUCs of 0.806, 0.717, and 0.727 for 1, 3, and 5 years, respectively. NPLOC4 expression was analyzed according to age, sex, AJCC stage, and TNM stage of the patients (Supplementary Fig. 2). Validation in the organization chip was substantially consistent with analysis results in TCGA database.

### Cox regression analyses of the TCGA database and TMA cohort

In TCGA cohort (Table 1), both univariate and multifactorial Cox regression analyses models, NPLOC4 expression and age were significantly associated with overall survival (OS). Most importantly, patients with LUSC have a shorter OS with an increase in NPLOC4 expression. Then verified with the TMA database (Table 2). In univariate Cox regression analysis models, age, TNM stage, AJCC stage, and NPLOC4 expression were significantly associated with OS (*p* < 0.05). In the multivariate Cox regression analysis, NPLOC4 expression was associated with OS (*p* < 0.05). Both TCGA and the TMA databases revealed a correlation between NPLOC4 expression and OS. NPLOC4 may be an independent prognostic factor for lung cancer.

**Table 1:** Univariate and Multifactor Cox regression analysis of OS in TCGA cohort.

Characteristics	Univariate analysis			Multivariate analysia		
	HR	95%CI	*P* value	HR	95%CI	*P* value
Age	1.0236	1.0045–1.0431	0.0153	1.0293	1.0094–1.0497	0.0037
Gender	1.3595	0.9441–1.9577	0.0988	1.3748	0.9515–1.9863	0.0900
Stage	1.2298	1.0261–1.4738	0.0251	1.1714	0.7606–1.8041	0.4728
T	1.2182	0.9984–1.4873	0.0525	1.1173	0.8231–1.5168	0.4768
N	1.1179	0.901–1.387	0.3113	0.9825	0.6602–1.4622	0.9308
M	2.4324	0.8977–6.5911	0.0804	1.5996	0.4447–5.7544	0.4720
NPLOC4	1.4202	1.0077–2.0017	0.0451	1.5159	1.0613–2.1653	0.0222

*OS* overall survival, *TCGA*: The Cancer Genome Atlas.

**Table 2:** Univariate and multifactor cox regression analysis of OS in TMA cohort.

Characteristics	Univariate analysis			Multivariate analysia		
	HR	95%CI	*P* value	HR	95%CI	*P* value
Gender	0.69	0.21–2.27	0.54	0.71	0.09–5.87	0.75
Age	1.05	1.01–1.1	0.02	1.04	0.97–1.12	0.25
Grade	0.76	0.35–1.66	0.49	0.87	0.24–3.16	0.83
Region	1.45	0.75–2.79	0.27	1.08	0.39–2.96	0.89
T	1.51	0.93–2.44	0.09	0.95	0.43–2.11	0.9
N	2.4	1.16–4.94	0.02	1.07	0.26–4.42	0.93
M	6.6	0.86–50.86	0.07	3.31	0.11–96.63	0.49
Stage	2.04	1.26–3.28	0.0035	1.77	0.59–5.35	0.31
PD-L1	0.69	0.34–1.38	0.29	0.94	0.34–2.6	0.9
CD8	0.24	0.0015–39.73	0.59	0.67	0.0016–275.96	0.9
NPLOC4	2.5	1.52–4.12	0.00032	2.56	1.18–5.55	0.017

*OS* overall survival, *TMA*:Tissue microarrays were engineered by Shanghai Outdo Biotech Company(Shanghai, China).

### Protein interaction networks and predicted signaling pathways

**Figure 3:**
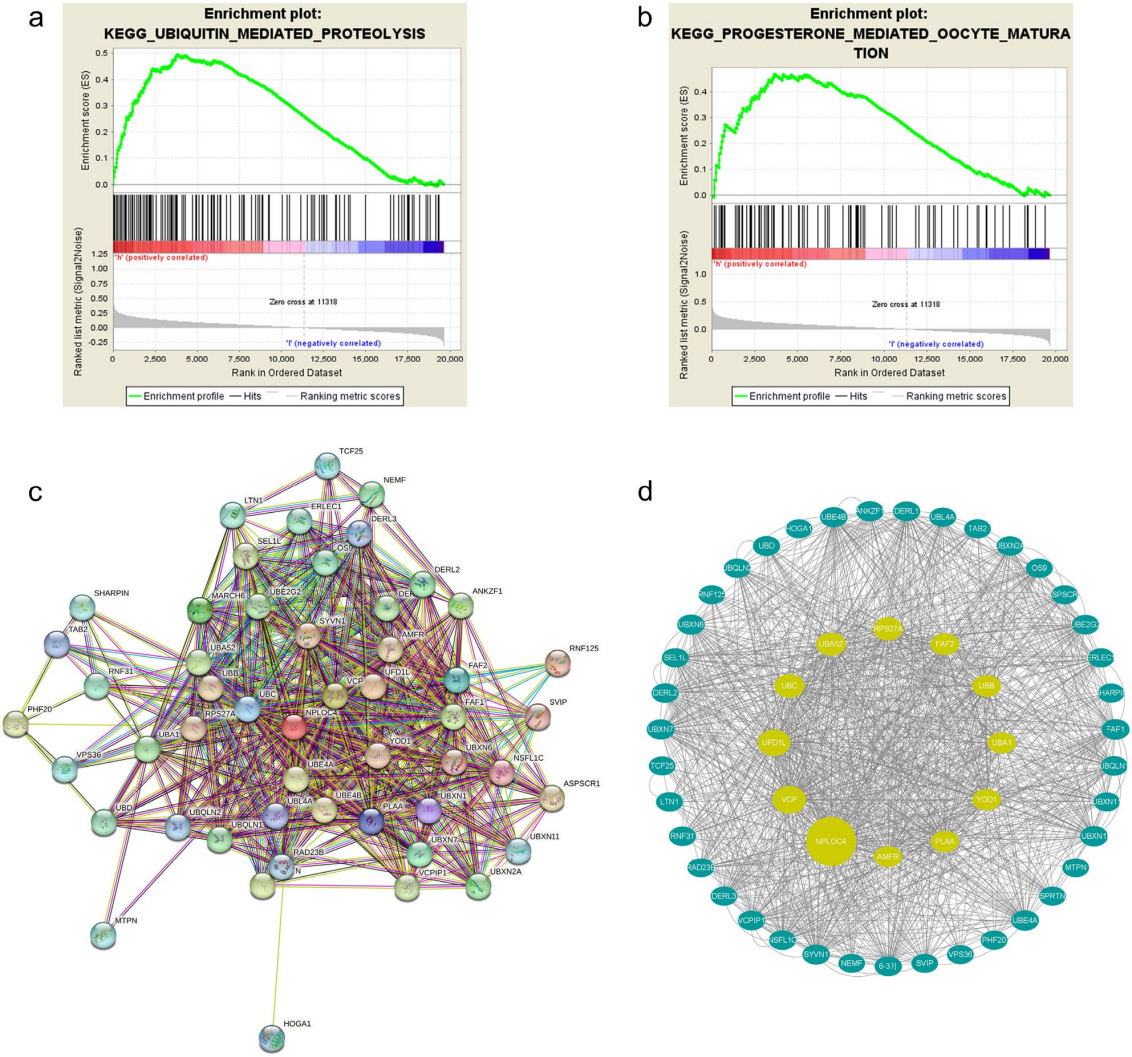
KEGG enrichment analysis of LUSC. (**a**) Ubiquitin-mediated proteolysis. (**b**) Progesterone-mediated oocyte maturation. (**c**) The PPI network of NPLOC4. A visual network of NPLOC4 and its co-expression genes. (**d**) Visual Analysis of STRING Protein Interaction Network.

Next, we used TCGA database to identify NPLOC4 pathway mechanisms that may have an impact on pulmonary squamous carcinoma prognosis. The results in TCGA dataset suggested that gene expression in the high-risk group was significantly enriched in ubiquitin-mediated proteolysis (Fig. 3a) and progesterone-mediated oocyte maturation (Fig. 3b). We used the STRING database to analyze the network of protein interactions associated with NPLOC4. Our screening criteria included a maximum of 50 interactors and a minimum interaction score of 0.4. The protein interaction network of NPLOC4 is shown in Fig. 3c. And quantitative analysis of the interaction network using cytoscape found that NPLOC4 is closely related ubiquitinated protein (Fig. 3d). Furthermore, Molecular Function (Gene Ontology) identified K48-linked polyubiquitin modification-dependent protein binding. We therefore hypothesize that NPLOC4 affects pulmonary squamous carcinoma prognosis via the K48-linked ubiquitin-mediated proteolytic pathway.

### Prediction of drug targets and pathway validation

**Figure 4:**
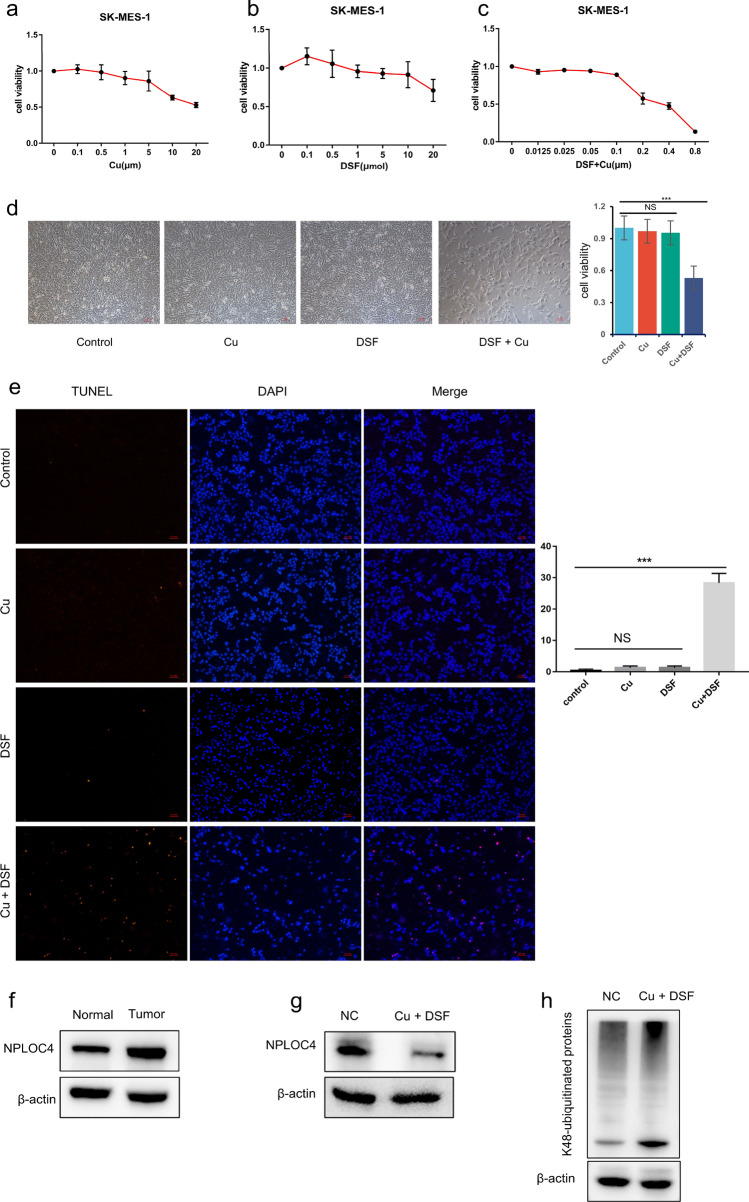
Disulfiram combined with copper increases cytotoxicity to lung squamous cells. SK-MES-1 cells treated with increasing concentrations of (**a**) DSF, (**b**) Cu, or (**c**) DSF + Cu for 24 h. (**d**) Morphological changes photographed under a microscope (100 × magnification) (Scale bar: 100 µm). SK-MES-1 cells were exposed to 0.2 μm Cu, 0.25 μm DSF or DSF + Cu (0.25 μm + 0.2 μm) for 24 h, (**e**) apoptosis observed with TUNEL and fluorescence microscopy (Scale bar = 100 µm). (**f**) NPLOC4 expression in normal BEAS-2B cells and SK-MES-1 cells. SK-MES-1 cells were exposed to DSF + Cu (0.25 μm + 0.2 μm) for 24 h, (**g**) NPLOC4 expression level, (**h**) K48-ubiquitinated protein expression level.

In our previous research, DSF + Cu was found to inhibit ubiquitin-mediated proteolysis by acting on NPLOC4, resulting in gastric cancer cell apoptosis^12^. However, this has yet to be demonstrated in lung cancer. First, we used Cu (Fig. 4a) and DSF (Fig. 4b) to verify the cytotoxic effect of the two drugs on SK-MES-1 cells. The CCK8 results showed decreased cell survival as the concentration of the two drugs increased, with 50% cell survival at 0.2 μM Cu and 0.25 μ M DSF. DSF combined with Cu had a higher cell inhibition rate than the single drugs in SK-MES-1 cells (Fig. 4c). Cell viabilities were 97% for Cu, 95% for DSF, and 53% for DSF + Cu (Fig. 4d). Apoptosis was detected using TUNEL kits, and we found that DSF + Cu-treated cells had the highest apoptosis rate among the four groups (Fig. 4e). When Cu was combined with DSF, NPLOC4 expression was inhibited compared with that in the control group (Fig. 4g). Compared with the control group, the DSF + Cu group exhibited increased K48-ubiquitinated protein expression (Fig. 4h). Furthermore, we found that NPLOC4 expression is higher in tumor cells than in normal cells (Fig. 4f).

### Correlation between NPLOC4 and tumor immunity

**Figure 5:**
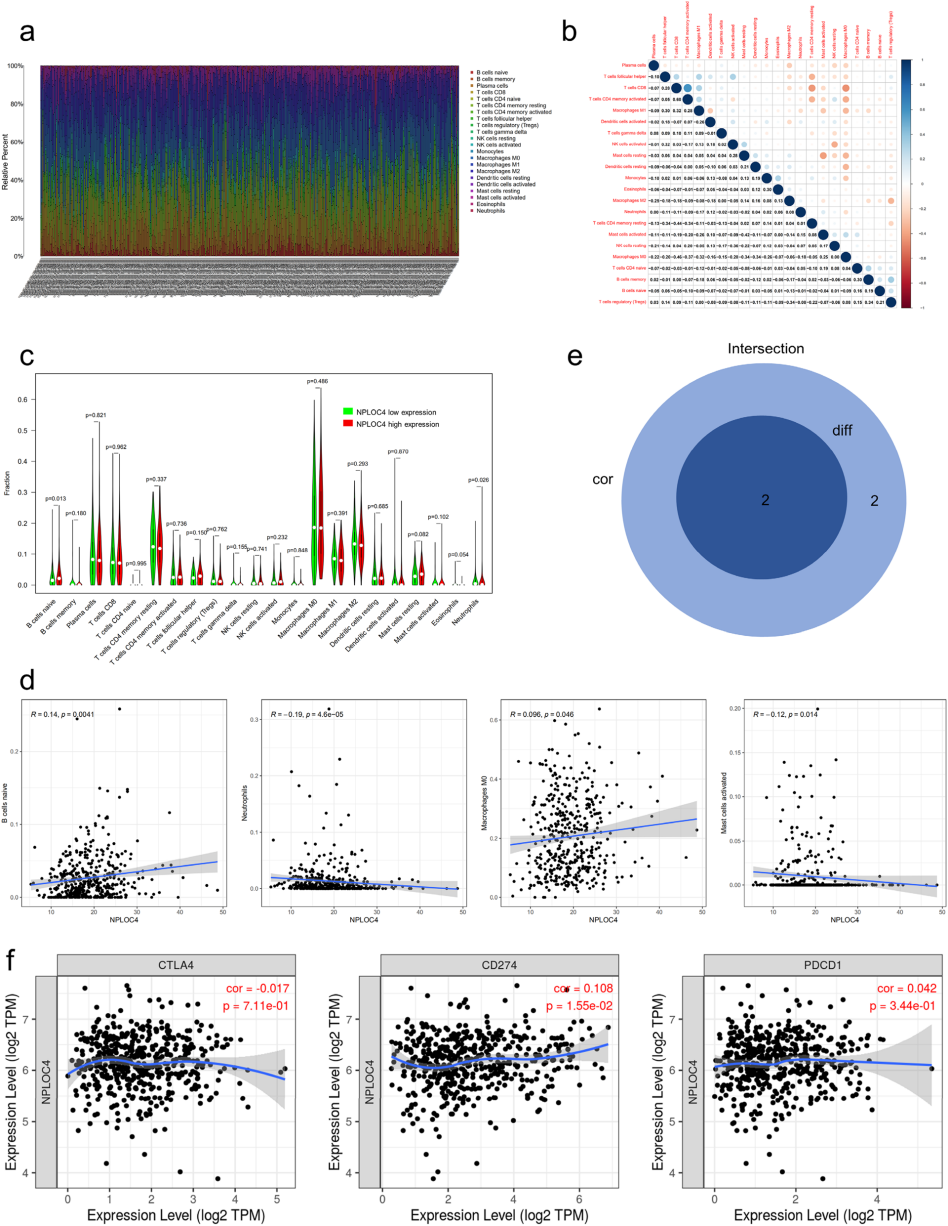
Immune cell infiltration in squamous lung cancer and correlation analysis. Significance tests using the Pearson’s coefficient. (**a**) The percentages of 22 TICs in LUSC tumor samples are shown. The column names represent sample ID. (**b**) Analysis of correlations between TICs. The shade of the circles in each square represents the degree of correlation between the two cells. The number in each square quantifies the correlation. (**c**) Violin diagram showing the differences in the proportions of the 22 immune cells between the high NPLOC4 expression group and the low NPLOC4 expression group. (**d**) Scatter plots representing the correlation between immune cells and NPLOC4 expression. (**e**) Venn plot showing two types of TICs with NPLOC4 expression codetermined by difference and correlation tests displayed in violin and scatter plots, respectively. (**f**) Correlation of NPLOC4 expression with three immune checkpoints analyzed using the TIMER database.

With the rise of immunotherapy, its role is extremely important in tumor treatment. At the end of the study, we analyzed the correlation between NPLOC4 expression and immune cells and immune checkpoints. We analyzed the relative proportions of 22 types of immune cells in LUSC samples from TCGA database (Fig. 5a) and constructed an immune cell proportion correlation heat map (Fig. 5b). Next, we evaluated the differences in immune cell proportions associated with high and low NPLOC4 expression (Fig. 5c) and the correlation between NPLOC4 expression and immune cells (Fig. 5d). We identified two types of cells associated with differential NPLOC4 expression, namely naive B cells and neutrophils (Fig. 5e). Using the TIMER database, we analyzed correlations between NPLOC4 expression and immune checkpoints CTLA-4, PD-L1, and PD-1 (Fig. 5f). Among these, PD-L1 expression exhibited the greatest correlation with NPLOC4 expression. NPLOC4 expression affects the immune microenvironment of LUSC. High NPLOC4 expression in LUSC also increased the proportion of naive B cells and decreased the neutrophil proportion. However, the positive correlation between PD-L1 and NPLOC4 expression in the analysis of the database might suggest that patients benefit more from immunotherapy.

## Discussion

In this study, we identified NPLOC4 as a prognostic biomarker for LUSC, with elevated NPLOC expression associated with shorter survival and poor prognosis. In addition, we found a positive correlation between NPLOC4 and PD-L1 expression. Finally, in LUSC, DSF combined with Cu might induce tumor cell apoptosis by inhibiting NPLOC4, which increases ubiquitinated protein accumulation.

NPLOC4 is an important cofactor of p97 and, together with ubiquitin fusion degradation 1 (UFD1), is involved in many p97-dependent processes^15,16^ such as ER-associated protein degradation. p97 is considered a potential cancer biomarker, associated with poor prognosis, increased likelihood of metastasis, or shorter OS in pancreatic cancer^17^, liver cancer^18^, breast cancer^19^, prostate cancer^20^, and lung cancer^21^. In pan-cancer analysis, the expression of NPLOC4 in lung adenocarcinoma differed significantly between cancer and paracancerous, as shown in Fig. 1a. We analyzed the effect of the expression of NPLOC4 in lung adenocarcinoma on the survival of patients, and found that there was no significant different survival between high and low groups (Supplementary Fig. 4). In lung adenocarcinoma, we considered that the presence of some gene hot-spot mutations affected the survival of patients with the expression of NPLOC4. In order to ensure the homogeneity of the study cohort, we selected lung squamous cell carcinoma for study. We found that NPLOC4 expression is associated with shorter OS, later AJCC staging, lymph node metastasis, and poor prognosis in LUSC. However, during the study, our research group found that in pancreatic adenocarcinoma, the group with high NPLOC4 expression had longer survival times (Supplementary Fig. 3), suggesting that the effect of NPLOC4 is specific to the tumor type.

In 1977, Lewison published the first clinical case report on DSF as an anticancer therapy. Skrott et al.^10^ analyzed medical data from 240,000 patients with cancer in Denmark and reported that patients with alcoholism treated with disulfiram consistently over a long period had a 34% reduction in mortality compared with those who did not receive disulfiram. Studies have shown that disulfiram exerts its antitumor effects through interaction with Cu^14,22–25^, and that DSF + Cu exerts antitumor effects by targeting ALDH-positive tumor stem cells^26,27^. However, further investigation indicated that DSF + Cu does not target ALDH^13^, but binds NPLOC4 and induce its aggregation, disabling the p97-NPLOC4-UFD1 pathway and increasing the accumulation of ubiquitinated proteins, leading to cell death^10,12,28,29^. This mechanism has been studied in several tumor types, including gastric cancer^12^, medulloblastoma^30^, and clear cell renal cell carcinoma^14^. Although the effects of DSF + Cu has been studied in non-small cell lung cancer^31,32^, no studies have been conducted to investigate NPLOC4 inhibition or the effect of DSF + Cu on LUSC. In this study, we found that DSF + Cu induces apoptosis in lung squamous carcinoma cells possibly by inhibiting NPLOC4, thereby increasing accumulation of ubiquitinated proteins. We also found a positive correlation between NPLOC4 and PD-L1 expression. DSF + Cu has been shown to upregulate PD-L1 expression, and the combination of DSF + Cu and anti-PD-1 antibody showed better antitumor efficacy than monotherapy in hepatocellular carcinoma^33^ and triple-negative breast cancer^34^. The combination regimen is therefore promising for treatment of patients with tumors, but further evidence of the efficacy is required.

Clinical trials of DSF have been conducted for multiple tumor types (https://clinicaltrials.gov/). A phase IIb clinical trial of non-small cell lung cancer (ClinicalTrials.gov NCT00312819) found increased survival in the combined DSF + chemotherapy group compared with the chemotherapy monotherapy group (10 months vs 7.1 months)^35^.

We believe that screening for high NPLOC4 expression levels may provide a greater survival benefit. Future studies to validate the antitumor effect of DSF in lung squamous carcinoma in animal experiments, as well as a larger clinical sample size to evaluate the effect of NPLOC4 expression on the prognosis of patients with LUSC, is required. This study identified a novel prognostic biomarker for patients with LUSC, as well as a new focus for targeted therapy. Furthermore, as the mutation rate of PD-L1 in lung squamous carcinoma is higher than that in lung adenocarcinoma^36^, this study provides a new proposal for combined immune-targeted therapy for lung squamous carcinoma.

### Supplementary Information

Supplementary Figure S1.

Supplementary Figure S2.

Supplementary Figure S3.

Supplementary Figure S4.

Supplementary Information 5.

Supplementary Information 6.

## Data Availability

The datasets generated during and analyzed during the current study are available in The Cancer Genome Atlas (TCGA) database (https://portal.gdc.cancer.gov/), UCSC Xena database (https://xena.ucsc.edu/), Oncolnc online database (http://www.oncolnc.org/), STRING (https://cn.stringdb.org/) and TIMER database (https://cistrome.shinyapps.io/timer/). The original contributions presented in the study are included in the article/Supplementary Material. Further inquiries can be directed to the corresponding author.
