# Supplementary figures and images for: NPLOC4 is a potential target and a poor prognostic signature in lung squamous cell carcinoma
Source: Sci Rep. 2023 Nov 22;13:20430. doi: 10.1038/s41598-023-47782-6 (PMC10665339; doi:10.1038/s41598-023-47782-6)

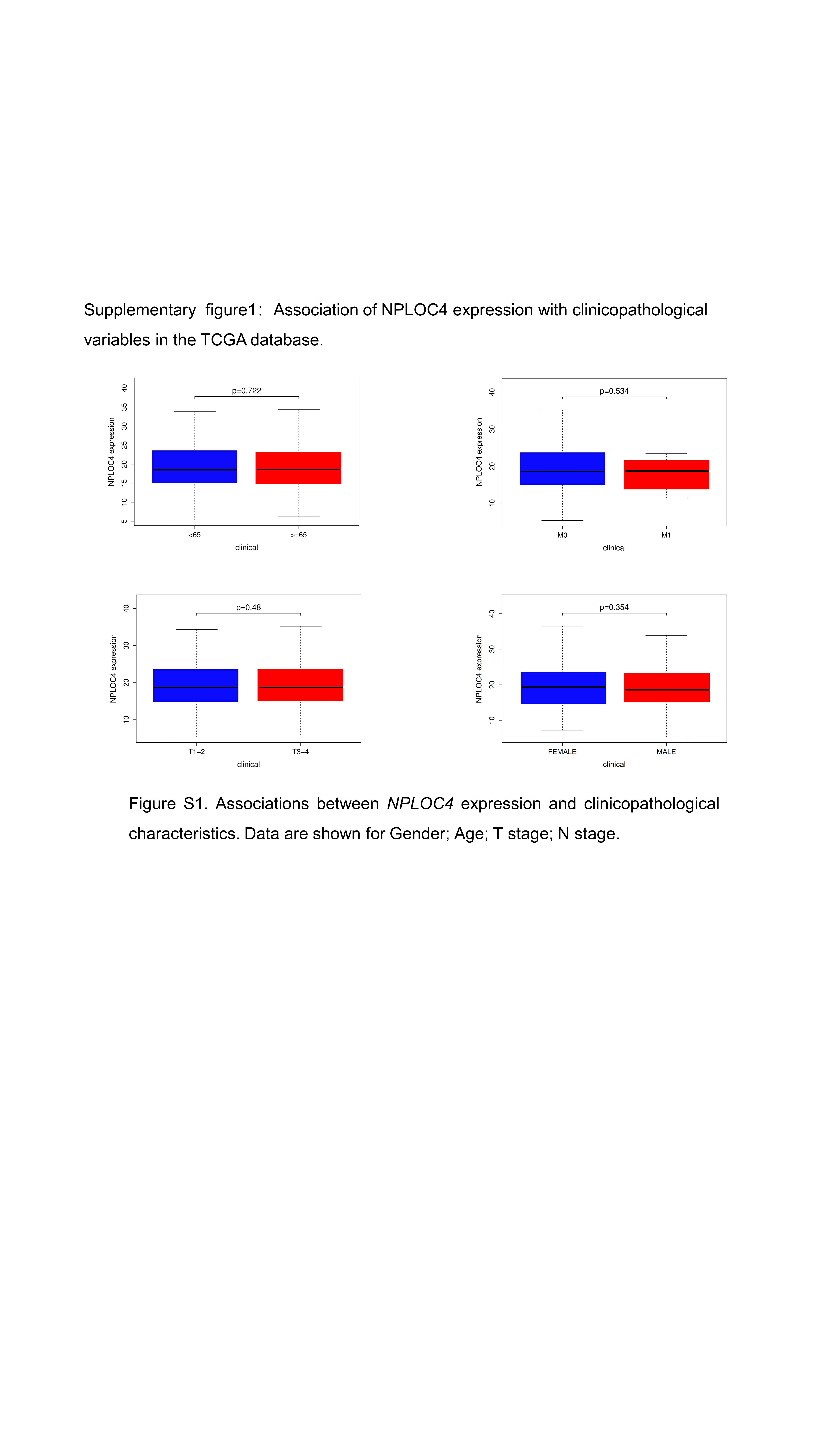

Supplement: Supplementary file 1 — Supplementary Figure S1. [file 41598_2023_47782_MOESM1_ESM.tif]

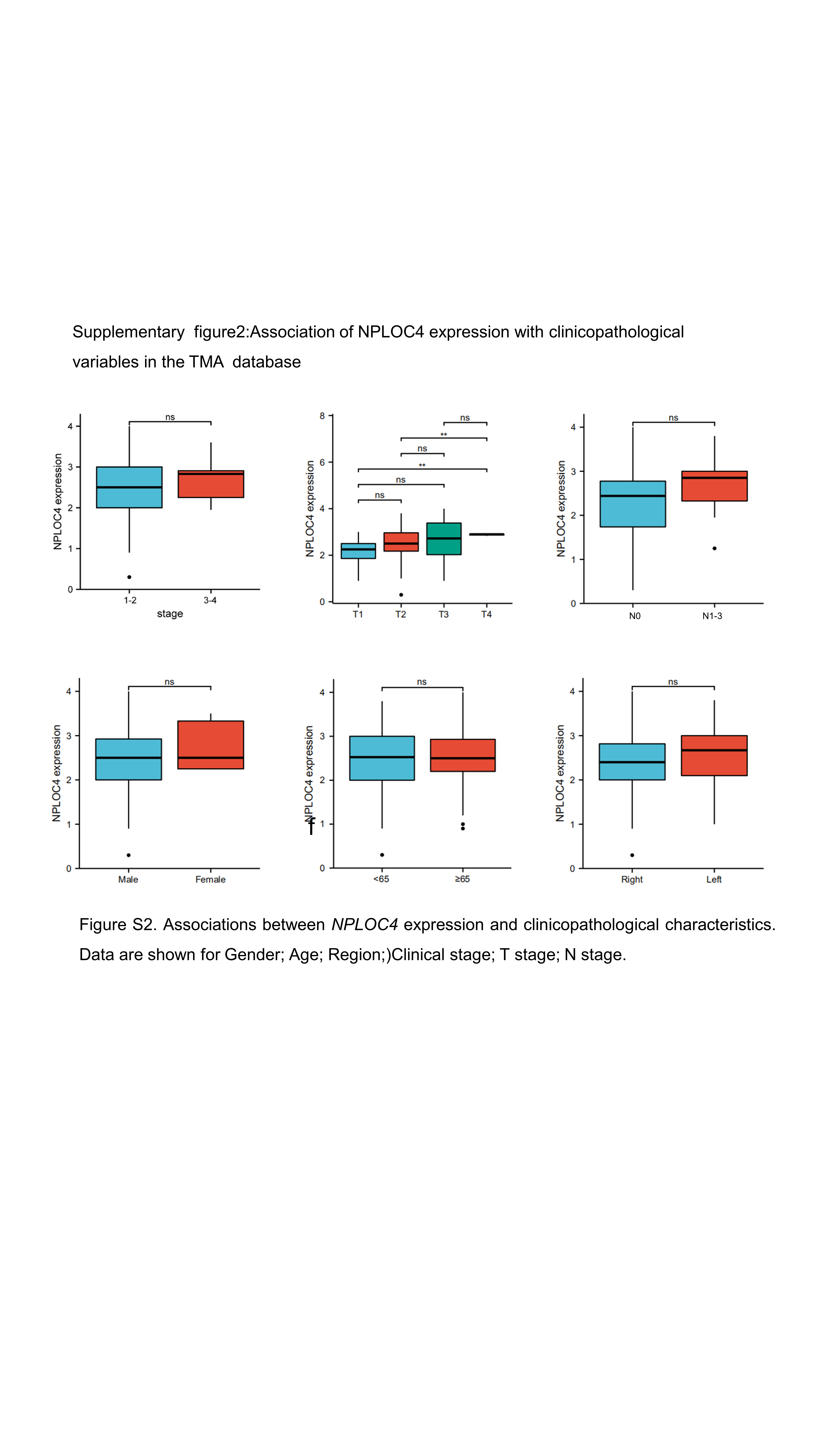

Supplement: Supplementary file 2 — Supplementary Figure S2. [file 41598_2023_47782_MOESM2_ESM.tif]

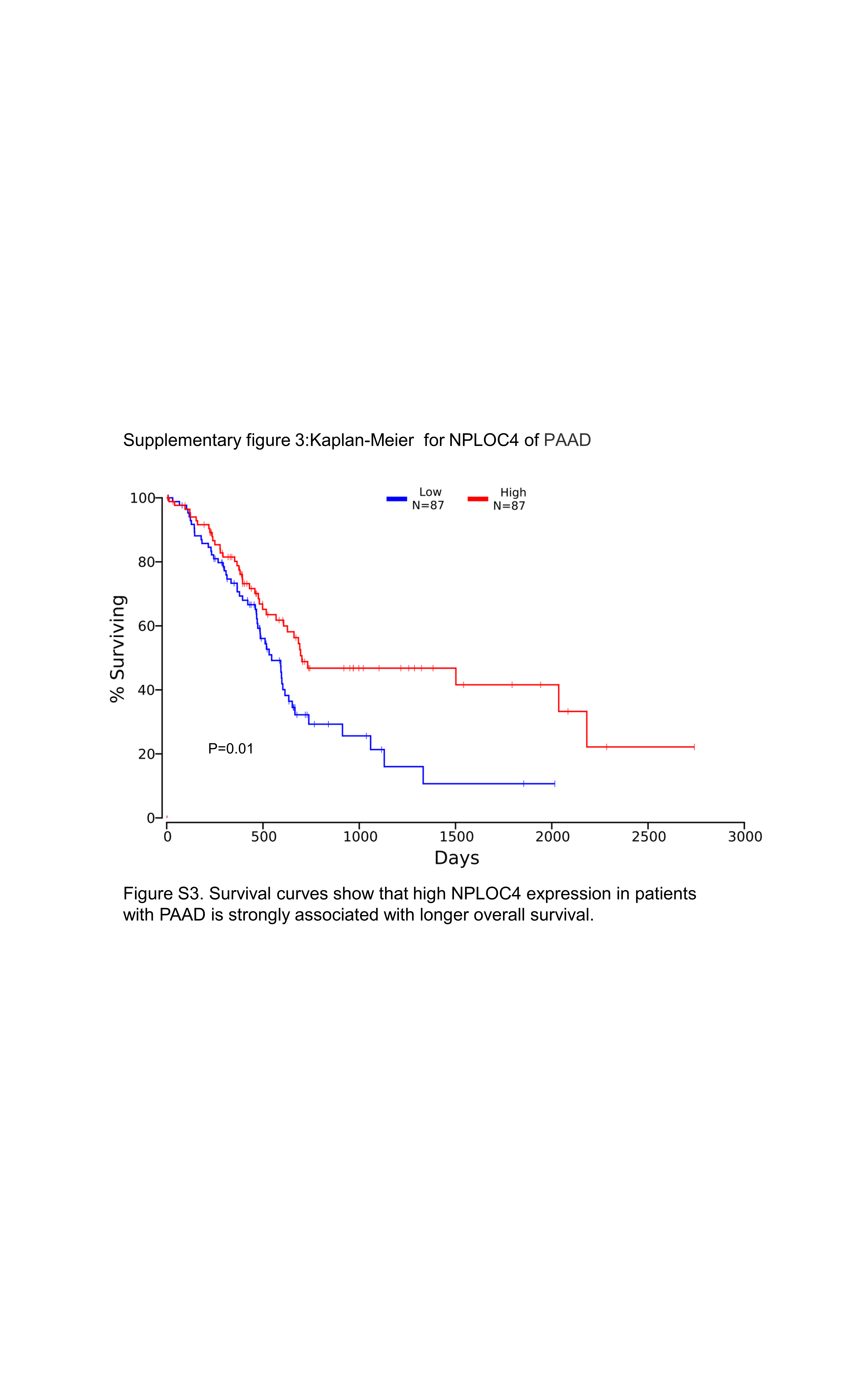

Supplement: Supplementary file 3 — Supplementary Figure S3. [file 41598_2023_47782_MOESM3_ESM.tif]

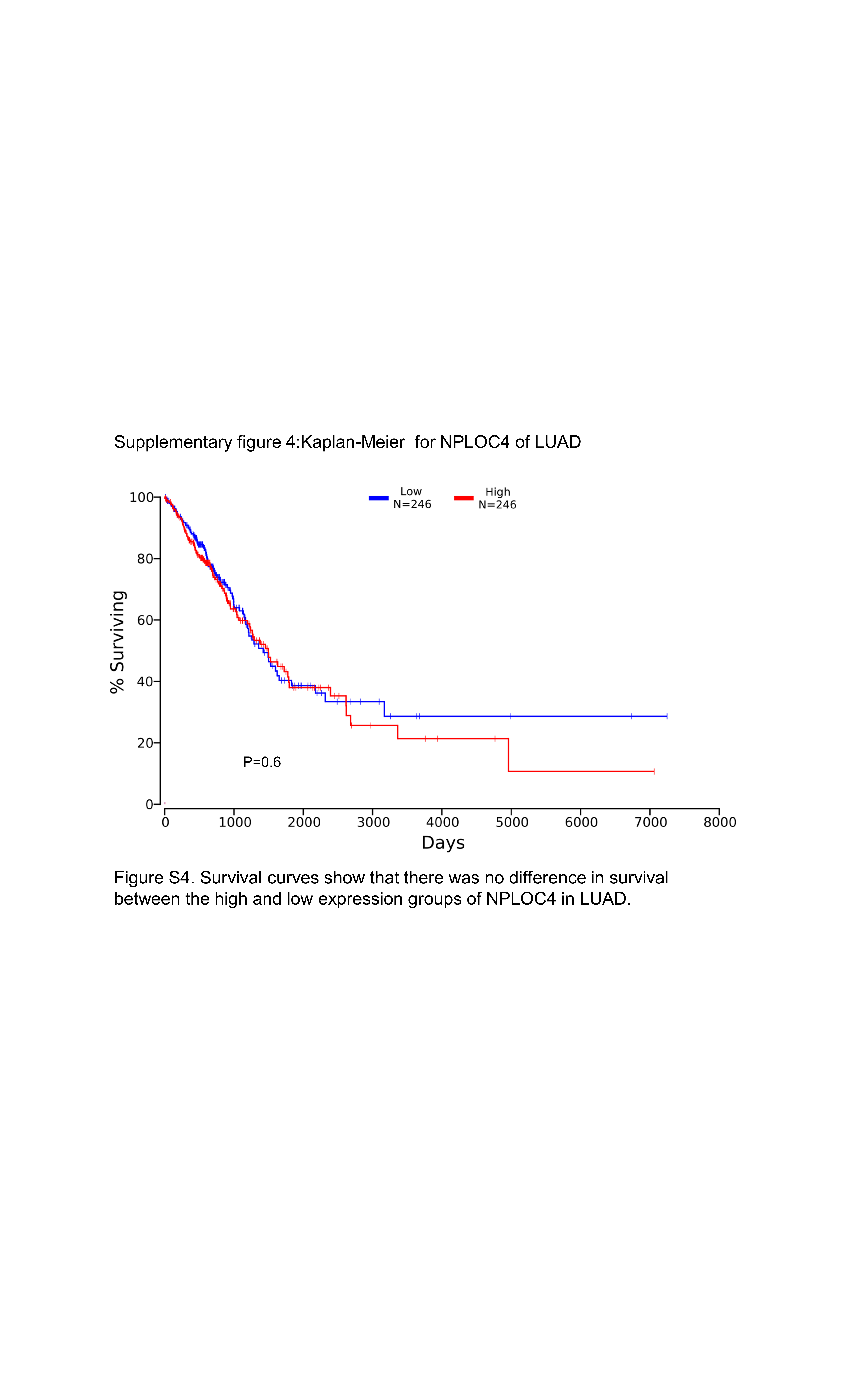

Supplement: Supplementary file 4 — Supplementary Figure S4. [file 41598_2023_47782_MOESM4_ESM.tif]

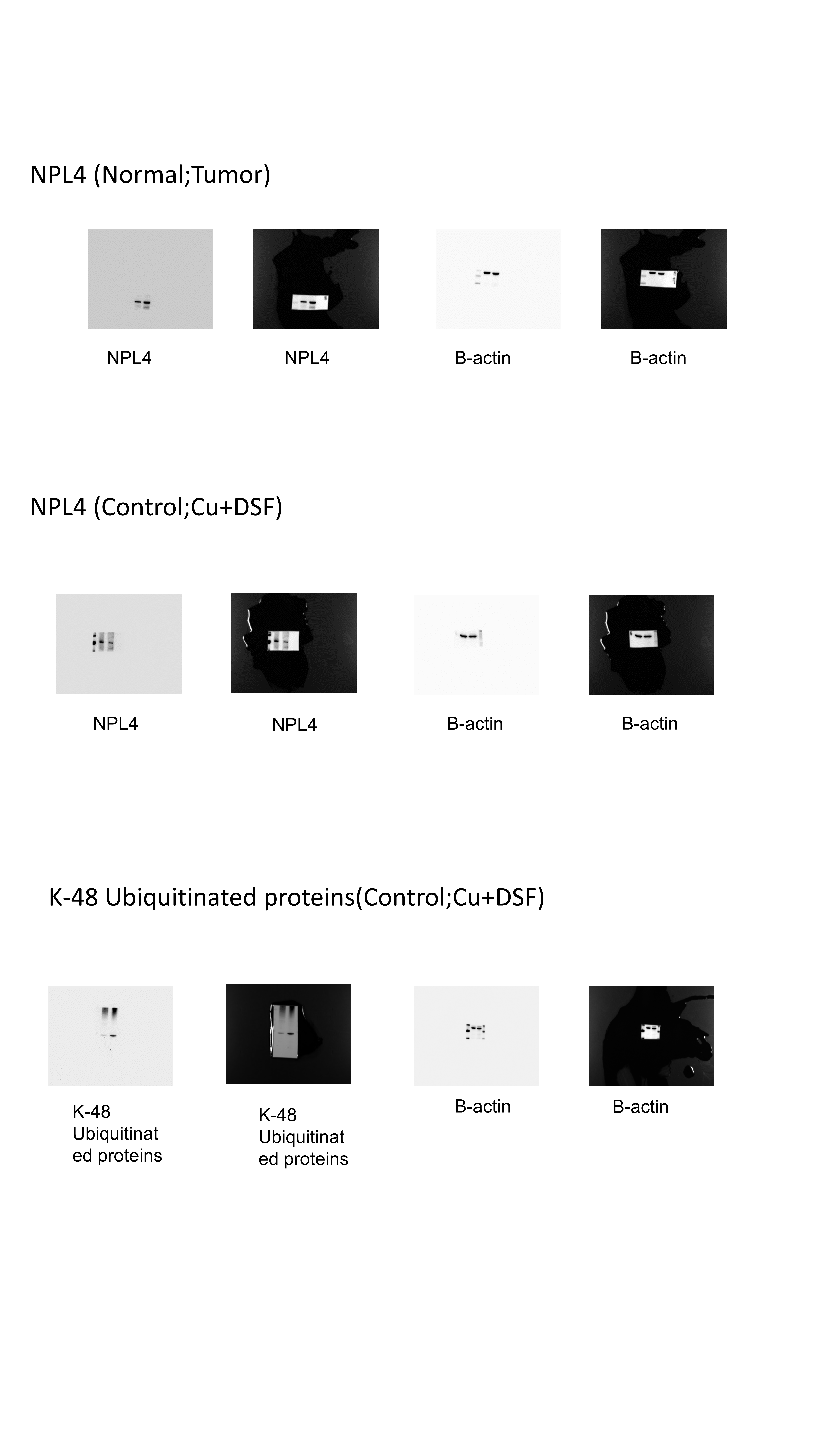

Supplement: Supplementary file 5 — Supplementary Information 5. [file 41598_2023_47782_MOESM5_ESM.tif]

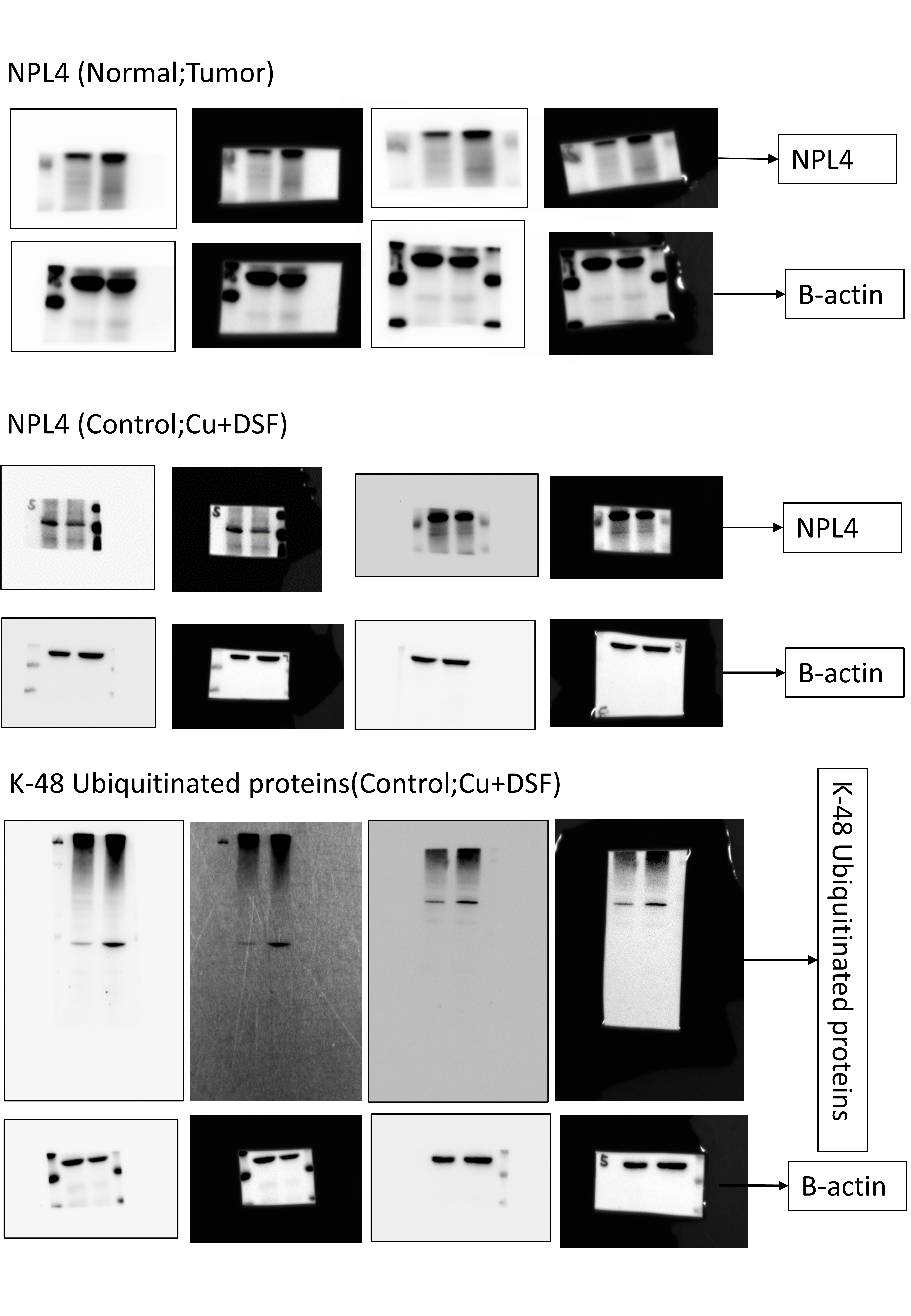

Supplement: Supplementary file 6 — Supplementary Information 6. [file 41598_2023_47782_MOESM6_ESM.tif]
